# LuffaFolio: A Multidimensional Image Dataset of Smooth Luffa

**DOI:** 10.1016/j.dib.2024.110149

**Published:** 2024-02-05

**Authors:** Md Ripon Sheikh, Md. Masudul Islam, Galib Muhammad Shahriar Himel

**Affiliations:** a Department of Computer Science and Engineering, Bangladesh University of Business and Technology (BUBT), Dhaka, Bangladesh; b Department of Computer Science, American International University-Bangladesh, Dhaka, Bangladesh; c Department of Computer Science and Engineering, Jahangirnagar University, Dhaka, Bangladesh; d Department of Physics, Jahangirnagar University, Dhaka, Bangladesh; e School of Computer Sciences, Universiti Sains Malaysia, 11800 USM Penang, Malaysia

**Keywords:** Smooth Luffa, Image dataset, Diseases classification, Luffa leaves, Image processing, Agriculture

## Abstract

This article introduces a comprehensive dataset designed for researchers to classify diseases in Luffa leaves, determine the grade of Luffa from Luffa images, and identify different growth stages throughout the year. The dataset is meticulously organized into three sections, each concentrating on specific facets of Luffa Aegyptiaca, commonly known as Smooth Luffa (Dhundol/). These images were captured in various village fields in Faridpur, Bangladesh. The sections include the assessment of Smooth Luffa quality, the identification of plant diseases, and the documentation of Luffa flowers. The dataset is divided into three sections, totaling 1933 original JPG images. The “Luffa Diseases” section features images of smooth Luffa leaves, depicting various diseases and unaffected leaves. Categories in this section encompass Alternaria Disease, Angular Spot Disease, Holed Leaves, Mosaic Virus, and Fresh Leaves, totaling 1228 JPG raw images. The “Flowers” category comprises 362 JPG raw images, showcasing different maturity stages in smooth Luffa flowers. Finally, the “Luffa Grade” section focuses on categorizing smooth Luffa into fresh and defective categories, presenting 343 JPG raw images for this purpose.

Specifications Table

**Table:** 

Subject	Computer Science
Specific subject area	Computer Vision, Pattern Recognition, machine learning, deep learning
Data format	Raw
Type of data	JPG Image
Data collection	This dataset is divided into 3 parts; Each part consists of smooth luffa grading, diseases, and flowers respectively. These images were captured from different village fields of Faridpur, Bangladesh. To construct the dataset, we have considered *Luffa Aegyptiaca* commonly known as Smooth Luffa (Dhundol/). This dataset consists of a total of 1933 JPG images; the dimension of each image is 1728 × 1728 pixels. The total size of the dataset is 1.45 GB. The dataset contains 3 folders: Luffa_Diseases, Flowers, and Luffa_Grade. The detail of the dataset: • **Luffa_Diseases**: This dataset contains leaves of smooth luffa representing various diseases along with non-affected ones. The categories of this dataset are Alternaria Disease, Angular Spot Disease, Holed Leaves, Mosaic Virus, and Fresh Leaves. A total of 1228 JPG raw images are presented in this folder. • **Flowers**: This dataset contains flowers of smooth luffa. There is only a single category of this dataset. A total of 362 JPG raw images are presented in this folder. These images represent various maturity stages of smooth luffa flowers. • **Luffa_Grade**: This dataset contains fresh and defective smooth luffa. The categories of this dataset are Fresh and Faulty Luffa. A total of 343 JPG raw images are presented in this folder. The dataset images were diligently taken from 2nd to 23rd October 2023.
**Data source location**	**Location:** Faridpur **Zone:** Binokdia **Country:** Bangladesh
**Data accessibility**	**Repository name:** Mendeley Data **Data identification number:** 10.17632/kwj9599z73.2 **Direct URL to data**: https://data.mendeley.com/datasets/kwj9599z73/

## 1 Value of the Data

• The dataset presents a comprehensive compilation of Luffa leaves affected by four distinct diseases, furnishing invaluable visual data for researchers engaged in the study of plant pathology. This facilitates the identification, detailed analysis, and formulation of effective management strategies for these diseases within Luffa crops.
• Including images representing two Luffa grade types, rotten and fresh, establishes a valuable resource for the evaluation of Luffa produce quality. Researchers and agricultural practitioners can leverage this dataset to construct models for quality assessment, thereby enhancing agricultural practices and market value.
• Applications and machine learning models developed with this dataset can significantly aid agricultural practitioners, including farmers, in disease detection and crop management, contributing to heightened yield and quality enabling farmers to avoid economic loss.
• This dataset is a valuable asset for data scientists and machine learning experts specializing in image classification and pattern recognition. It stands as a benchmark dataset for the development and testing of algorithms related to disease identification, quality grading, and flower stage recognition within agricultural contexts.
• Regression algorithms can be applied to the Luffa Flower data to assess the maturity or growth stages level of Luffa. Flower images help farmers and agricultural experts assess the overall health and vigor of the plant. Additionally, these images can be used for yield prediction models, enabling farmers to anticipate harvest quantities based on observed flowering patterns.

## 2 Background

Smooth Luffa, scientifically known as Luffa Aegyptiaca, is a warm-season vegetable with distinctive lobed leaves and elongated green fruit. Its mature, dried fruit transforms into natural sponges used for exfoliation. The plant produces large, eye-catching yellow flowers, representing its reproductive cycle and different growth stages. Smooth Luffa cultivation faces challenges from diseases like Alternaria Disease and Mosaic Virus, requiring effective management for quality fruit production. Beyond agriculture, Luffa serves in traditional medicine and sustainable personal care [1], with its sponges offering a natural alternative to synthetic materials. This dual role makes Smooth Luffa significant in both agriculture and everyday life, fostering research and sustainable practices.

In the past few years, there has been a growing utilization of leaf images for the identification of diseases and infestations [2,3]. Additionally, leaf images have proven valuable for the recognition of plant species [4], [5], [6], [7]. From these studies, it is evident that leaf images serve a multifaceted purpose, encompassing disease recognition, plant species identification, and the differentiation between healthy and unhealthy plants.

## 3 Data Description

**Fig. 1:**
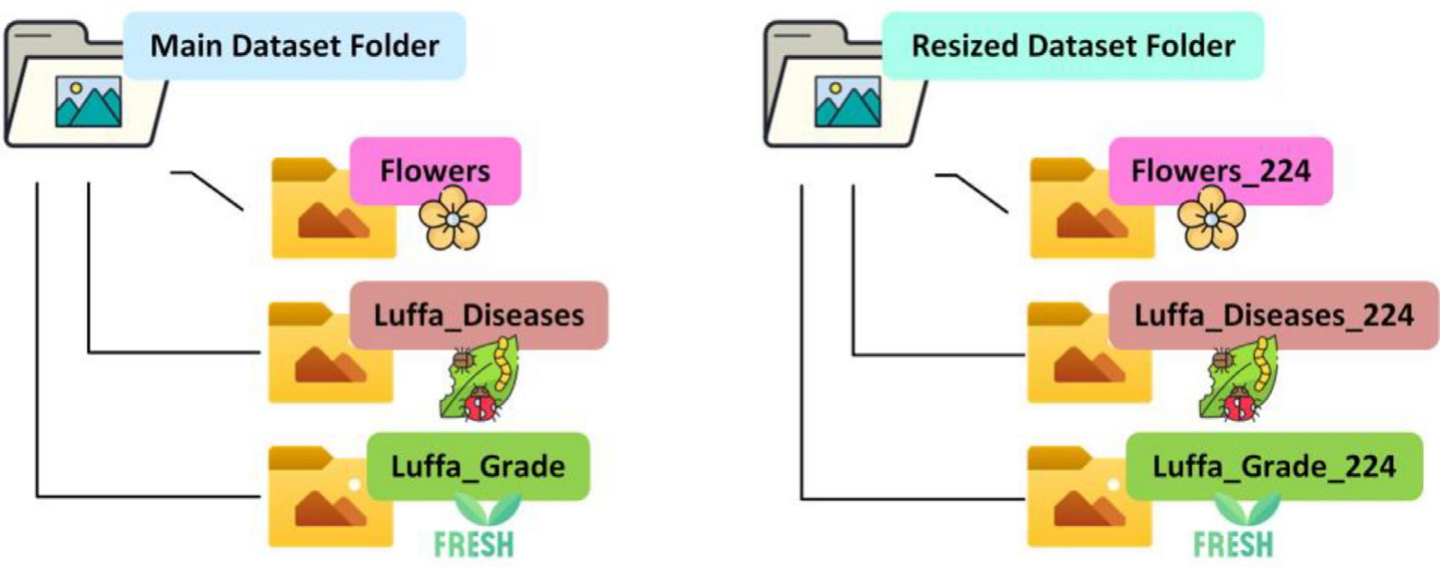
Smooth Luffa dataset folder structure.

**Fig. 2:**
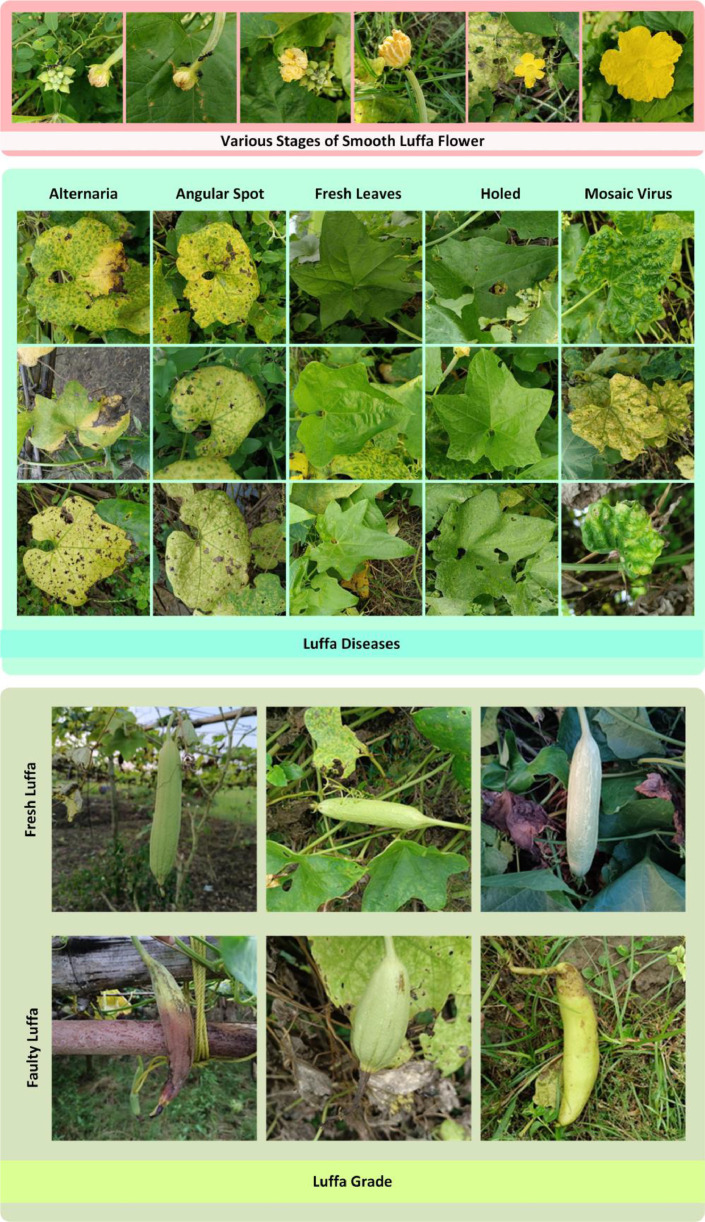
Image examples of datasets.

In our research paper, we introduced a raw and resized (224 × 224 pixels) image dataset that encompasses three main components: Luffa Diseases, Flowers, and Luffa Grade. Each component is subdivided into distinct sub-folders. The “Luffa Disease” category includes five sub-folders—Alternaria, Angular Spot, Fresh, Holed, and Mosaic Virus—comprising a total of 1,228 raw JPG images. These images are consistently formatted at a resolution of 1728 × 1728 pixels, resulting in a substantial file size of 945 MB. The "Flowers" section presents a total of 362 raw JPG images, all set at a uniform resolution of 1728 × 1728 pixels, resulting in an initial file size of 203 MB. In the “Luffa Grade” category, there are two sub-folders: Fresh and Faulty, featuring a total of 343 raw JPG images, also consistently formatted at 1728 × 1728 pixels, resulting in a file size of 343 MB. Our dataset's folder structure is visually represented in Fig. 1, while the dataset generation process is illustrated in Fig. 2. Table 1 describes the visual features of the Luffa dataset. This dataset is conveniently available through the Mendeley Data [8], stored in three separate zip files: 'Luffa_Diseases.zip,' 'Flowers.zip,' and 'Luffa_Grade.zip' inside the ‘Original Dataset’ folder and ‘Smooth_Luffa_Dataset_224.zip’ inside ‘Resized Dataset’ folder. Our dataset can be utilized from various viewpoints or dimensions to identify different aspects of Luffa, including diseases, growth stages, and freshness status (whether healthy or rotten).

**Table 1:** Visual features of the luffa dataset.

**Luffa Diseases**	
**Alternaria**	Alternaria leaf blight symptoms appear as dark brown to black irregularly shaped lesions on leaf blades and petioles. Spots are initially surrounded by a yellow margin and often begin on the older leaves. Leaves can be killed when spots grow together. Lesions that develop on petioles may kill entire leaves.
**Angular Spot**	Lesions on the foliage begin as water-soaked spots that later turn gray or tan. Spots may initially develop a yellow halo. As the affected tissue dries, the internal tissue may fall out, giving the leaf a tattered appearance. The lesions are delimited by veins, giving them an angular shape.
**Holed**	Shot hole is a leaf disease that produces brown spots on plant foliage that eventually dry out, fall, and leave multiple holes in the leaf.
**Mosaic Virus**	Mosaic symptoms are variable but commonly include irregular leaf mottling (light and dark green or yellow patches or streaks). Leaves are commonly stunted, curled, or puckered; veins may be lighter than normal or banded with dark green or yellow.
**Fresh Leaves**	Typically, these leaves are characterized by a vibrant green coloration, reflecting a healthy and vigorous state of the plant. The leaf surface is generally smooth and unblemished, with well-defined margins and a consistent leaf shape. The texture is often glossy, conveying a level of hydration and vitality. Veins are prominently visible, branching out in an organized pattern across the leaf surface.
**Luffa Grade**	
**Fresh Luffa**	They typically have a vibrant green color, a firm and plump texture, smooth skin without blemishes or discolorations, and a well-defined, uniform shape. The appearance is indicative of the Luffa's prime state, suggesting optimal ripeness and quality for consumption or use.
**Rotten Luffa**	Rotten Luffa typically includes a change in color from the usual green to brown or yellow, a soft and mushy texture, the potential presence of mold or fungal growth, and an unpleasant odor. The skin may appear wrinkled or discolored, and the overall appearance is indicative of decay and deterioration. These visual cues help in identifying and distinguishing rotten Luffa from its fresh and healthy counterpart.
**Luffa Flowers**	
**Flowers**	**Bud Stage:** Small and tightly closed, often with a distinct green hue. **Blooming Stage:** Petals begin to unfurl, revealing the flower's vibrant color, typically yellow. The flower expands, and the petals become more visible. **Mature Stage:** Fully opened flower with fully extended petals. The color is intense, and the bloom is at its maximum size. **Fading Stage:** Petals may start to wilt or change color, indicating the end of the flowering period.

## 4 Experimental Design, Materials and Methods

**Fig. 3:**
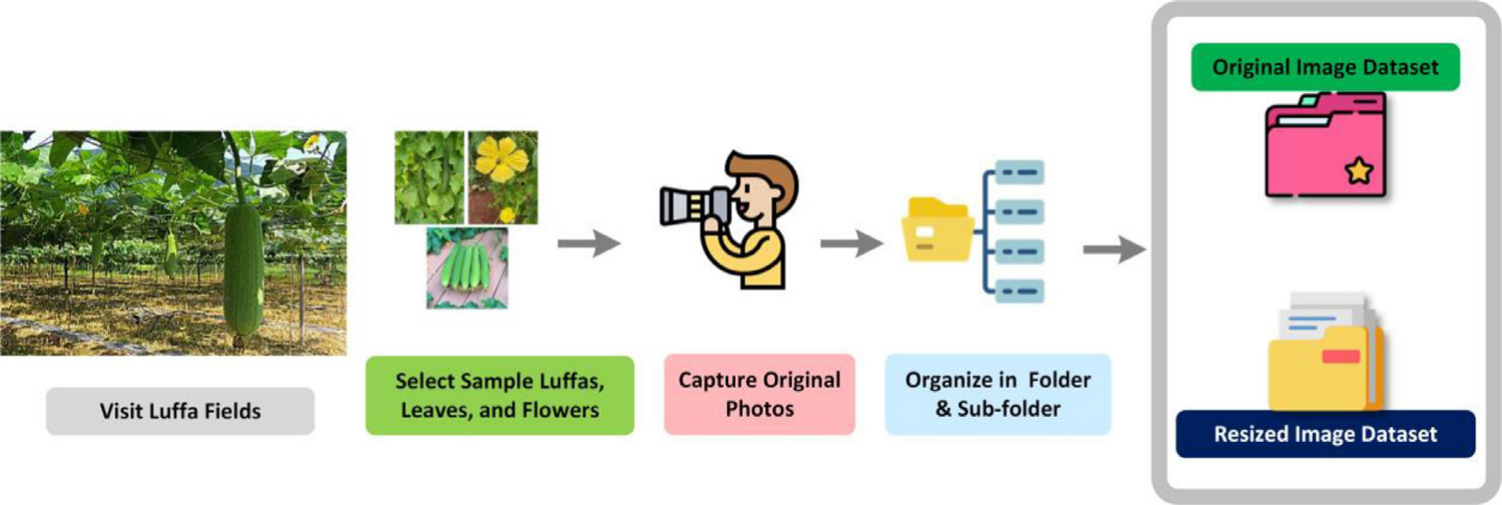
Workflow for the smooth Luffa dataset collection.

The process of acquiring images for the Smooth Luffa dataset adhered to a specific workflow, which is visualized in Fig. 3. In this data collection method, we employed a systematic approach to ensure a diverse and representative selection of Smooth Luffa images within our dataset. The dataset encompasses three distinct sub-datasets: Luffa Flowers, Luffa Grade/Quality (comprising Rotten and Fresh Luffa), and Luffa Diseases, which includes five categories (Alternaria, Angular Spot, Holed, Mosaic Virus, and Fresh) depicting the health of Luffa Leaves. The data collection process began in various fields across different villages within the Binokdia region of Faridpur, Bangladesh. To maintain diversity and equal representation among these categories, we followed a non-uniform distribution approach. Images for each category were collected using a random selection process. Luffa specimens, whether representing flowers, Luffa quality, or disease categories, were chosen randomly from within their respective groups. We captured the images using a smartphone camera under the guidance of an expert in the agricultural field. After capturing images, they were promptly transferred from the smartphone's memory to an external hard drive for safe storage. To maintain the systematic approach, we proceeded to collect images for the next category only after clearing the smartphone's memory of the previously captured category. This process was diligently followed until we obtained a comprehensive representation of the dataset, covering the full spectrum of Luffa, from various stages of flower maturity to Luffa quality and the presence of specific leaf diseases. This method ensured a well-rounded and diverse dataset, ready for further analysis and research.

Table 2 shows the statistical view of our datasets.

**Table 2:** Statistics of the smooth Luffa dataset.

Types	Categories	Number of original images	Total images
***Luffa Grade***	Fresh	183	**343**
	Faulty	160	
***Luffa Diseases***	Alternaria	270	**1228**
	Angular Spot	238	
	Holed	222	
	Mosaic Virus	241	
	Fresh	257	
***Luffa Flowers***		362	**362**

### 4.1 Camera specification

To capture these images, we have used the VIVO V25 5G smartphone camera with LED flash, HDR, and panorama features. Its triple camera has the following specification: 64 MP, f/1.8, (wide), 0.7 µm, PDAF, OIS**,** 8 MP, f/2.2, 16 mm, 120° (ultrawide), 1/4", 1.12µm**,** 2 MP, f/2.4, (macro).

## CRediT authorship contribution statement

**Md Ripon Sheikh:** Investigation, Software, Validation, Formal analysis, Methodology, Resources, Data curation, Writing – original draft, Visualization. **Md. Masudul Islam:** Project administration, Conceptualization, Methodology, Formal analysis, Resources, Writing – original draft. **Galib Muhammad Shahriar Himel:** Project administration, Supervision, Conceptualization, Methodology, Formal analysis, Resources, Writing – original draft, Writing – review & editing.

## Data Availability

LuffaFolio: A Multifaceted Luffa Aegyptiaca Image Dataset (Original data) (Mendeley Data).
